# PB.53: Are ethnic minorities more likely to develop triple-negative breast cancer? A systematic review

**DOI:** 10.1186/bcr3553

**Published:** 2013-11-08

**Authors:** R Limbada, A Slater, AK Jain

**Affiliations:** 1 De Montfort University, Leicester, UK; 2 The Nightingale Centre and Genesis Prevention Centre, Wythenshawe Hospital, Manchester, UK

## Introduction

Triple-negative breast cancer (TNBC) accounts for 10 to 15% of diagnosed breast cancers worldwide. TNBC is negative for oestrogen receptor, progesterone receptor and human epidermal growth factor receptor 2 expression, and is associated with a poor prognosis. Our study aims to systematically review the possible link between various minority ethnic groups and development of TNBC.

## Methods

A systematic review of carefully selected studies was carried out with search of relevant articles in Medline, Science Direct and PubMed from 2005 up to 2013 with studies specifically aimed at TNBC. In the UK, receptor data have not been consistently recorded and involve smaller sample sizes. Hence, the relevant articles include ethnic groups from Asia Pacific and USA. Keywords were: triple negative, TNBC, ethnicity, incidence, prevalence. The data from all relevant articles were combined to give a sufficiently large sample size.

## Results

Complete receptor data were available for 72,763 cases, which constitute the sample group. On the available ethnicity data these were divided into five groups: Caucasian (*n *= 50,797), Black (*n *= 4,969), Asian (*n *= 6,909), Hispanic (*n *= 9,898) and other (*n *= 190). Overall there were 9,887 TNBC cases in the sample group with Caucasian (*n *= 5,956), Black (*n *= 1,341), Asian (*n *= 842), Hispanic (*n *= 1,732) and other (*n *= 16). Hence, overall 13.59% of all diagnosed breast cancers were TNBC with 11.73% in Caucasian, 26.99% in Black, 12.19% in Asian, 17.5% in Hispanic and 8.42% in other women.

## Conclusion

Overall minority ethnic women have higher incidence of TNBC than Caucasian women with resultant poor prognosis. With rising minority ethnic population in the UK the overall number of TNBC will rise. Further research is required into the reasons for this.

